# Editorial: Brain abnormalities due to genetic alterations or developmental exposure to environmental factors, volume II

**DOI:** 10.3389/fnins.2024.1389145

**Published:** 2024-07-11

**Authors:** Kazuhiko Sawada, Atsushi Yoshiki, Shigeyoshi Saito

**Affiliations:** ^1^Department of Nutrition, Faculty of Medical and Health Sciences, Tsukuba International University, Tsuchiura, Japan; ^2^Experimental Animal Division, RIKEN BioResource Research Center, Tsukuba, Japan; ^3^Division of Health Sciences, Department of Medical Physics and Engineering, Graduate School of Medicine, Osaka University, Suita, Osaka, Japan; ^4^Department of Advanced Medical Technologies, National Cerebral and Cardiovascular Center Research Institute, Suita, Osaka, Japan

**Keywords:** dystonia, *Hspa8*, mTORC1, very preterm, tobacco smoking, fetal alcohol spectrum disorders, maternal separation, valproic acid

The brain develops structurally and functionally under the influences of genetic and environmental factors. A Research Topic previously published in *Frontiers in Neuroscience* titled “Brain abnormalities due to genetic alterations or developmental exposure to environmental factors” (Sawada et al., 2022) contains articles that focused on “models for neurodevelopmental disorders” and “impacts of particular molecules and/or genes in the brain maldevelopment” in mammals including humans. These studies have deepened our insights into the pathogenesis of autism spectrum disorder and attention deficit hyperactivity disorder caused by environmental factors such as endocrine disruptors, epigenetic dysregulation and parental smoking. Successively, the current Research Topic “Volume II” has been initiated, resulting in the acceptance of eight papers, comprising one review article and seven original articles.

Three articles on this Research Topic focused on genetic alterations in neurodevelopment. A review article by Fan et al. summarizes the pathogenesis of a neurological disorder, named “DYT-*TOR1A* dystonia,” which carries mutations in the *TOR1A* gene encoding for an adenosine triphosphatase. The motor symptoms of patients with DYT-*TOR1A* dystonia are characterized by involuntary muscle contractions of the trunk and hands and an inability to complete fine, complex finger movements. These may be involved in the disorganization of the basal ganglia-cerebellum-thalamus-cortex circuitry. Notably, deletion of the *TOR1A* gene alters brain morphogenesis by the disarrangement of apical radial glia in the ventricular zone, which give rise to cerebral cortical neurons (Ossola and Kalebic, 2022), in mouse fetuses on embryonic day 14.5. An original article by Tanaka et al. characterized the histopathological changes of the brain and spinal cord in a newly established autosomal recessive mutant rat, F344-*kk/kk*, that exhibited abnormal hind limb gaits with ataxia. In F344-*kk/kk* rats, several axonal spheroids were identified mainly in the dorsal horn of the spinal cord, cerebellar white matter, and accessory cuneate and dorsal column nuclei of the medulla oblongata, diagnosed with neuroaxonal dystrophy. Positional cloning identified a missense mutation in the gene for *Hspa8* (heat shock protein family A (Hsp70) member 8), which is located on rat chromosome 8. Moreover, analysis of *Hspa8* mutant gene knock-in rats using genome editing revealed that the *Hspa8* gene is responsible for neuroaxonal dystrophy. An original article by Nguyen et al. evaluated the role of the mechanistic target of rapamycin complex 1 (mTORC1) pathway in cortical malformation and seizures during neurodevelopment in mice. Eukaryotic initiation factor 4E binding protein 1 (4E-BP1) is a mTORC1-downstream translational repressor. *In utero* electroporation was performed to express a constitutively active 4E-BP1 mutant (4E-BP1^CA^) either in apical radial glia that gives rise to the cerebral cortical neurons (Ossola and Kalebic, 2022) or in migrating cortical neurons using a conditional expression system. The expression of 4E-BP1^CA^ in both neurogenetic stages of cortical neurons partially improved mTORC1-induced cortical malformation. However, the effect of 4E-BP1^CA^ expression on seizures varies at the differential stages of corticoneurogenesis. Furthermore, mTORC1-related behavioral seizure severity was enhanced when 4E-BP1CA was expressed in apical radial glia. Conversely, the severity was attenuated when 4E-BP1CA was conditionally expressed in migrating cortical neurons.

Five original articles in this Research Topic focused on the influences of various environmental factors on brain development during the prenatal and postnatal periods. Liu et al. investigated the differences in brain white matter microstructure and network connectivity between 43 term-equivalent infants born at gestational weeks 27 to 32 (very preterm; VP) and 40 infants born at gestational weeks 37 to 44 (full-term) using conventional magnetic resonance (MR) imaging and diffusion tensor imaging (DTI) in humans. In some white matter structures including the corpus callosum, a significant difference between VP infants and full-term infants was observed in DTI-defined fractional anisotropy, which evaluated the degree of white matter development in terms of myelination or integrity of the fiber tracts (Pecheva et al., 2018). Poor brain network connectivity was also observed in VP infants. These abnormalities in white matter development in VP infants may be associated with perinatal factors such as gestational age at birth, weight, gestational hypertension, infection and/or activity, pulse, grimace, appearance, respiratory score. Kobus et al. conducted a questionnaire survey on the involvement of parental environment quality in offspring migraines in 80 male and 266 female human participants. Maternal tobacco smoking and traumatic events during pregnancy were associated with migraines in adulthood. Fraize et al. quantified the thickness and length of the corpus callosum extracted from T_1_-weighted anatomical MR images using an automated, normalization-free, fast method-based spectral analysis in infant-to-adolescent human subjects with fetal alcohol spectrum disorders (FASD) ranging from fetal alcohol syndrome (FAS) to non-syndromic forms (NS-FASD). Changes in the shape of the corpus callosum unrelated to the size of the brain were characterized by global excessive shortening with excessive thinning of the splenial part in participants with FAS and by excessive thinning of the isthmus additionally in individuals with FASD. Irie et al. investigated the influence of maternal separation on the development of the medial prefrontal cortex (mPFC) in rats. Rat infants were separated from their mothers twice daily, 3h each during postnatal days 2 to 20. As a result, they exhibited a selective decrease in parvalbumin expression in interneurons in the ventromedial mPFC on postnatal day 35, disturbing the excitatory/inhibitory balance. Such maternal separation-induced neurodevelopmental alterations may be involved in attenuating social recognition (Ohta et al., 2020). Notably, the reduced expression of parvalbumin improved to the levels observed in mother-reared control rats, when the rats were exposed to the enriched environment, followed by maternal separation (from postnatal day 21). Kamiya et al. reported a novel progenitor, named “internal granular progenitor (IGP)” that appeared transiently in the developing cerebellum of ferrets but was not observed in that of rodents. In the ferret cerebellum, IGPs may be a secondary source of granular neurons that are distinct from the external granular precursors. Valproic acid administration experiments revealed the epigenetic regulation of granular neurogenesis in IGPs.

The articles on this Research Topic have demonstrated various approaches for quantitatively investigating the abnormal development of the brain and spinal cord at the macro level such as conventional MR imaging and DTI, and at the micro level, such as stereology for estimating the neuron number and gene expression analysis. We hope that these papers will help readers improve their understanding of and gain insights into the field of neurodevelopment and provide suggestions for designing research plans for the advancement of this research field.

## Author contributions

KS: Writing – original draft, Writing – review & editing. AY: Writing – review & editing. SS: Writing – review & editing.
